# Solifugae of Canada

**DOI:** 10.3897/zookeys.819.25166

**Published:** 2019-01-24

**Authors:** Paula E. Cushing, Jack O. Brookhart

**Affiliations:** 1 Denver Museum of Nature & Science, Department of Zoology, 2001 Colorado Boulevard, Denver, Colorado 80205, USA Denver Museum of Nature & Science Denver United States of America

**Keywords:** biodiversity assessment, Biota of Canada, camel spiders, solifugids

## Abstract

The Solfugae fauna of Canada includes three known species: *Eremobatesdocolora* Brookhart and Muma, *E.scaber* (Kraepelin), and *Hemerotrechadenticulata* Muma. It is expected that as many as four additional species may be found in Canada. Only one Barcode Index Number is currently known from Canadian specimens.

The arachnid order Solifugae, commonly known as camel spiders, wind scorpions, or sun spiders, is a relatively small order with over 1100 described species, about 200 of which occur in North America (Harvey 2003, Brookhart and Brookhart 2006, Cushing et al. 2015). The order has been documented from the western Canadian provinces ranging from southwestern Saskatchewan to southern British Columbia. This corresponds generally to the Canadian Prairies and Western Interior Basin ecozones. Currently three species, *Eremobatesdocolora* Brookhart and Muma from Alberta and Saskatchewan, and *E.scaber* (Kraepelin) and *Hemerotrechadenticulata* Muma from British Columbia, have been recorded from Canada, all in the family Eremobatidae (Dondale 1979, Holmberg and Buckle 1992, Brookhart and Brookhart 2006) (Table 1). Dondale (1979) reported *E.gladiolus* Muma and Holmberg and Buckle (1982) added *E.pallipes* (Say), *E.scaber* (Kraepelin) and the genera *Eremochelis* and *Hemerotrecha* from Canada. Subsequently, *E.gladiolus* was synonymized with *E.scaber* by Brookhart and Cushing (2004), and Holmberg and Buckle (1992) determined that their report of *E.pallipes* and *Eremochelis* was based on misidentification.

**Table 1.** Census of Solifugae in Canada.

Taxon	No. species reported in Dondale (1979)	No. species currently known from Canada^1^	No. BINs^1^ available for Canadian species	Est. no. undescribed or unrecorded species in Canada	General distribution by ecozone^2^	Information sources
Eremobatidae	1	3	1	4	Western Interior Basin, Prairies	Dondale 1979, Holmberg and Buckle 1982, 1992, Brookhart and Brookhart 2006

^1^Barcode Index Number, as defined in Ratnasingham and Hebert (2013). ^2^See figure 1 in Langor (2019) for a map of ecozones.

Four undescribed species, two *Eremobates* and two *Hemerotrecha*, have been proposed from Canada (Holmberg and Buckle 1992), although these have not been formally described. These were collected from dry grassland habitats in Saskatchewan, Alberta and British Columbia (Brookhart and Brookhart 2006). These records are the basis for the estimate of four additional species remaining to be described from Canada (Table 1).

Presently, there are six specimens in the Barcodes of Life Data (BOLD) System from British Columbia, represented by one Barcode Index Number (BIN) (Table 1). These specimens are identified in BOLD as *Eremobatesgladiolus*, a junior synonym of *E.scaber*.

The low species diversity of this order of arachnids in Canada can be attributed to the lack of suitable habitats and climatic conditions in these northern latitudes for a group adapted to dry, warm, xeric conditions with open, sandy soils (Punzo 1998). Solifugae are best adapted to desert and semi-desert environments and are not well adapted to cold or temperate regions (Cloudsley-Thompson 1977), although at least one species, *Uspallatapulchra* Mello-Leitão, 1938 (Mummuciidae), has been reported from a high elevation site (3670 m) in Chile (Muma 1971). Future work on this order should focus on obtaining DNA barcode data from all taxa including the suspected new species.
